# Effects of Rugby Training Exposure on Knee-Jerk Reflex Magnitude: A Feasibility Study

**DOI:** 10.7759/cureus.104337

**Published:** 2026-02-26

**Authors:** Jamie Benson, Benjamin D Gompels, Ilektra Epanomeritaki, Alagu Subramanian, Stephen McDonnell, Hugh Matthews

**Affiliations:** 1 Department of Physiology, Development and Neuroscience, University of Cambridge, Cambridge, GBR; 2 Division of Trauma and Orthopaedics, University of Cambridge, Cambridge, GBR; 3 Department of Trauma and Orthopaedics, Addenbrooke's Hospital, Cambridge University Hospitals NHS Foundation Trust, Cambridge, GBR; 4 Faculty of Medicine, Edinburgh University, Cambridge, GBR; 5 Division of Trauma and Orthopaedics, Cambridge University Hospitals NHS Foundation Trust, Cambridge, GBR

**Keywords:** neuromuscular adaptation, patellar reflex, rugby, tendon reflex, training load

## Abstract

Background

This study aimed to quantify the patellar reflex and assess how rugby training level influences reflex magnitude, asymmetry, and sensitivity, using a novel and accessible method for patellar reflex quantification.

Methods

A novel force transducer and ankle-mounted accelerometer were used to measure raw acceleration, which was converted to lower-limb angular velocity in 64 male participants from four rugby player groups (control, amateur, semi-professional, and professional). Each participant underwent 20 reflex trials per leg. Reflex magnitude was derived from peak angular velocity. Asymmetry and sensitivity were assessed using repeated-measures ANOVA and distributional analyses.

Results

Reflex magnitude decreased progressively with increased rugby training (p<0.0001). No significant asymmetry was observed between dominant and non-dominant legs (p=0.411). Reflex sensitivity was also lower in professional players compared to controls (p<0.0001).

Conclusion

By developing a novel force transducer and accelerometer, this study presents an accessible and accurate method for reflex quantification that, with further development, may be of clinical interest. This study demonstrated a progressive diminution in patellar reflex responses with increased exposure to rugby training. This is indicative of training-dependent reflex adaptation, which may result from central and/or peripheral plasticity. Overall, a combination of factors likely causes the reflex changes observed with rugby training. However, a deeper understanding of these effects could aid injury prevention among elite athletes of both sexes across various sports and inform clinicians during medical examinations.

## Introduction

The knee-jerk reflex is a monosynaptic spinal reflex that counteracts sudden stretching of the quadriceps muscle [1]. The patellar tendon tap is detected by mechanosensitive stretch receptors in the muscle spindle, which excite Ia-afferent neurons. These neurons connect to motoneurons in the spinal cord, initiating reflex contraction in the quadriceps and homologous muscles, thereby evoking the knee-jerk response [2]. The reflex opposes rapid changes in quadriceps length, counteracts unexpected loads, and may aid force production during sprinting, maintain posture, or regulate muscle compliance [3-5].

Routine examination of the knee-jerk reflex is clinically significant, as abnormalities may indicate neurological pathology. Classifications of patellar tendon reflexes have previously been examined [6]. Diminished reflexes result from disorders of the peripheral nervous system (PNS), which may include dysfunction of sensory or motor axons, as well as central nervous system (CNS) pathology. In contrast, hyperreflexia is associated exclusively with CNS dysfunction, such as damage to the primary motor cortex or corticospinal pathways [7].

In athletes, the stretch reflex is modulated. Initial exposure affects reflex magnitude; for instance, training diminishes the Achilles tendon and patellar reflexes in ballet dancers [8]. Broadly, power-based sports reduce reflex magnitude, whereas endurance sports enhance it compared to untrained subjects [9,10]. Rugby, as a sport, requires both strength and endurance and straddles this binary division of endurance and power [11,12].

Asymmetrical training can differentially modulate reflex responses [13]. The asymmetrical effects of long-term training on muscle size in sports that involve unilateral muscle loading are well-documented [14,15]. However, asymmetrical reflex modulation is less well-documented. Modulation of spinal stretch reflex has been demonstrated in highly trained endurance runners, with muscle-specific effects observed between the soleus and tibialis anterior under pre-activation conditions [16]. 

In rugby players, there is an asymmetry in anthropometric and functional measurements, as bone, muscle, and fat mass differ between the dominant and non-dominant sides and lean muscle mass increases with regular training [17]. However, it is unknown whether asymmetries in reflex magnitude coexist as a result of these differences.

Previous studies have shown that tendon reflexes are significantly reduced in elite rugby players compared to untrained controls; however, these studies have been methodologically limited [18]. In one study, contrary to the clinical standard, each tendon was tapped only 2-4 times, rather than 5-6 times, which reduced the study's validity. Additionally, reflexes were graded subjectively, making the study susceptible to bias [19]. Another study found no asymmetries in the patellar reflexes of rugby players between their dominant and non-dominant legs [20]. However, the participants' average weight (83.4 kg) was notably low for a professional cohort, which undermines validity.

Currently, reflex assessments in clinical practice are qualitative and subjective, and the lack of a quantitative approach leads to high variability in clinicians' assessments [21]. Therefore, this feasibility study aims to investigate reflex magnitude in subjects with varying exposure to rugby training (amateur, semi-professional, and professional) using a novel force transducer and accelerometer. The primary outcome was to examine whether playing level affected the magnitude of reflexes.

## Materials and methods

Participants were recruited directly to local university and club rugby squads (Cambridge University Men's Blues Rugby, Cambridge Rugby Union Football Club (RUFC), and Harlequins Rugby Football Club (RFC)) and through campus advertisements for university students for the control group. Inclusion criteria required participants to be male adults actively training at the specified competitive level (amateur, semi-professional, or professional) or not engaged in structured sport (control group) and to be able to undergo reflex testing; individuals with an acute lower-limb injury preventing testing were excluded. 

**Figure 1:**
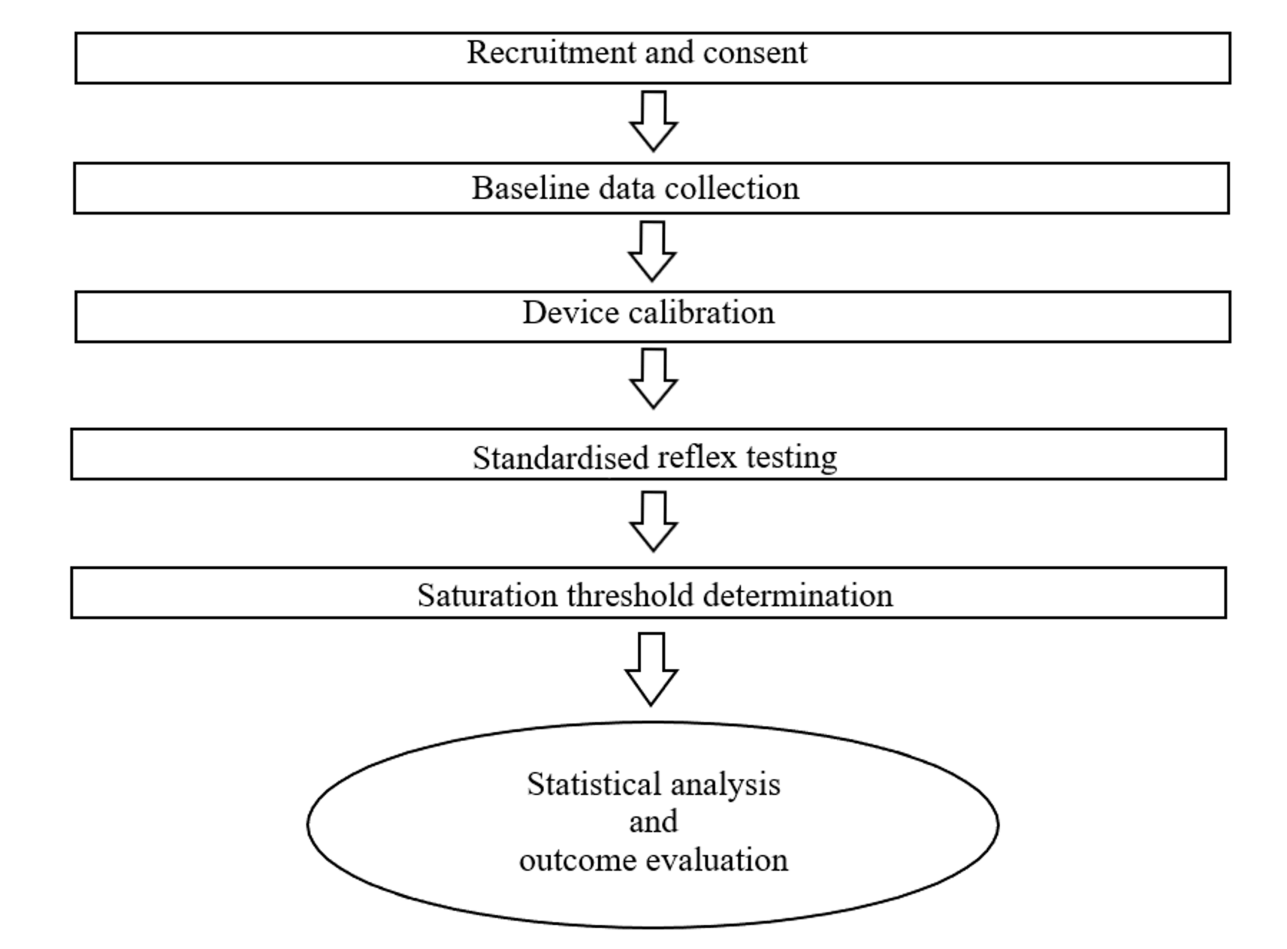
Flowchart of the experimental process from consent to analysis and outcome evaluation

A total of 64 men were recruited from the control, amateur, semi-professional, and professional groups. The demographic characteristics of each group are documented. The Human Biology Research Ethics Committee at the University of Cambridge granted ethical approval (approval number: HBREC.2022.27). The participants completed consent forms before testing. The experimental process is shown in Figure 1.

Quantification of the patellar reflex 

Measuring Stimulus Force 

To quantify the force of the blow evoking the reflex, a strain gauge, namely, the FX29 Compact Compression Load Cell (TE Connectivity, Galway, Ireland), contained in a custom housing 3D-printed from polylactic acid, was placed in contact with the patellar tendon and struck with a clinical tendon hammer. Placing the strain gauge over the tendon, rather than within the hammer, minimised the 'false strike' rate during each recording. To derive the magnitude of the force applied from each voltage reading, the device was calibrated under static and dynamic conditions, using known masses and collisions with a standardised transducer, respectively. 

Measuring the Acceleration of the Lower Leg 

A three-axis accelerometer GY-61 ADXL335 (Analog Devices, Wilmington, Massachusetts, United States) was employed to measure the linear acceleration of the lower leg [22]. The accelerometer, housed in a 3D-printed unit, was held to the back of the ankle with an elastic strap and aligned with the reflex movement. The novel force transducer and ankle-mounted accelerometer were purpose-built and integrated with established data acquisition systems (AD Instruments PowerLab and LabChart 7) to enable the accurate capture of stimulus force and reflex acceleration.

Experimental protocol 

Participants wore shorts to facilitate access to the knee and removed their footwear to minimise the effects of inertia on reflex magnitude. A questionnaire was developed to collect data on age, dominant leg, playing position, and injury history (see Appendices). Subjects sat relaxed on a bench with their knees at 90 degrees of flexion. An elastic strap secured the accelerometer to the right calf, at the superior edge of the malleoli, ensuring the measurement axis aligned with the reflex plane. The distance between the strap and the patella was recorded. A strain gauge was placed over the right patellar tendon. The reflex was elicited using a clinical tendon hammer on the device's force plate. 

Participants kept their eyes closed to avoid predicting the tendon hammer strike. To prevent unintentional Jendrassik manoeuvres, which might unevenly amplify responses, subjects relaxed their hands on the bench nearby. Wires hung loosely to minimise microphonic electrical interference noise [22,23].

**Figure 2:**
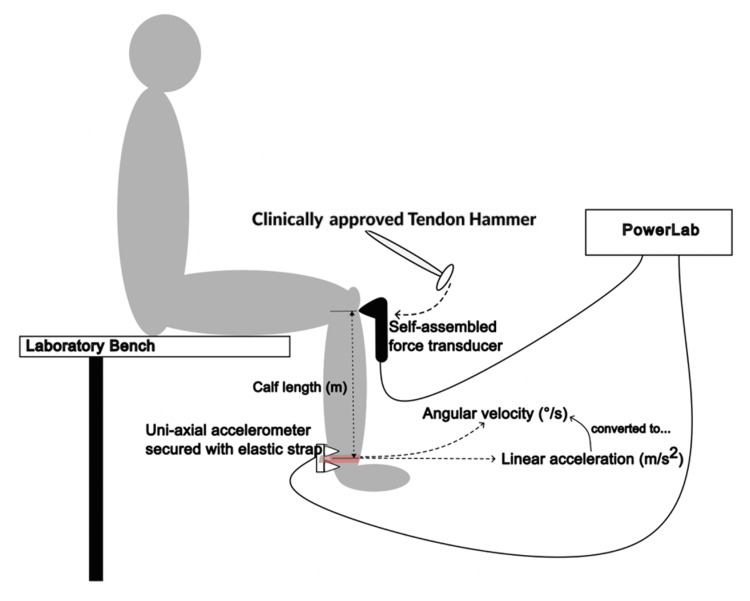
Diagram illustrating the experimental setup The participant sat on the edge of a lab bench. An accelerometer was attached to the back of the ankle, and a force transducer rested on the patellar tendon. The transducer recorded the force of the tendon hammer strike, while the accelerometer measured the resulting linear acceleration of the ankle.

A single investigator conducted all tests. Each subject completed 20 successful trials, discarding non-optimal strikes (mishits). No additional hits occurred until all secondary swinging had ceased, thus preventing any response influence. The protocol was replicated for the left leg. The experimental setup is illustrated in Figure 2. 

To identify the stimulus magnitude at which reflexes reach a maximum, 80 reflexes were elicited at varying forces and plotted as angular velocity against stimulus force. The initial protocol did not clearly demonstrate saturation, likely due to muscle fatigue, habituation, and experimenter variability. A revised protocol was therefore used: reflexes were recorded in sets of 10, each followed by a seven-minute rest, and the sequence was repeated eight times. This produced clearer evidence of saturation, and a sigmoidal fit identified a threshold for maximal reflex elicitation at 35 N.

Data processing 

Data were processed in MATLAB (version 24.1 (R2024a), The MathWorks, Inc., Natick, Massachusetts, United States) to extract peak force and acceleration data. The angular velocity traces, calculated by the integration of each acceleration trace, were manually scrutinised, and peaks derived from noise were excluded. The mean of the top 10 peak velocities per leg was used to derive velocity magnitude; a stimulus force threshold confirmed reflex response saturation. Analysing only the top 10 reflexes reduced variability. Linear velocity was manually converted to angular velocity.

Statistical analyses were conducted using RStudio (Version 2023.12.1+402, Posit PBC, Boston, Massachusetts, United States). To compare the groups, a two-way repeated-measures ANOVA with post hoc comparisons was employed. A two-sample Kolmogorov-Smirnov test was used to investigate distributional differences in reflex sensitivity between the control group and the professional group. Diagnostic plots and tests, including the Shapiro-Wilk and Levene's tests, validated the assumptions.

## Results

Demographics of the patient cohort 

The study involved 64 male subjects from the control, amateur, semi-professional, and professional groups, with 16 recruited from each group. The mean age for each cohort is shown in Table 1; the semi-professional group had the highest mean age. Training frequency increased with playing level. 

**Table 1:** Summary of subject demographic data The table shows the number of subjects, mean age (±standard deviation), source of subjects, and training frequency of each group. RUFC: Rugby Union Football Club; RFC: Rugby Football Club

Group	Number of subjects	Mean age in years (±SD)	Source of subjects	Training frequency (sessions per week)
Control	16	21.44±3.759	University students not engaged in sports	0
Amateur	16	22±2.828	Cambridge University Men's Blues Rugby squad	4-6
Semi-professional	16	28±3.347	Cambridge RUFC (second division of national rugby)	6-8
Professional	16	20.81±2.167	Harlequins RFC (top division of national rugby; includes international players)	8-10
Total	64	23.06±4.182		

Raw acceleration and derived velocity traces 

**Figure 3:**
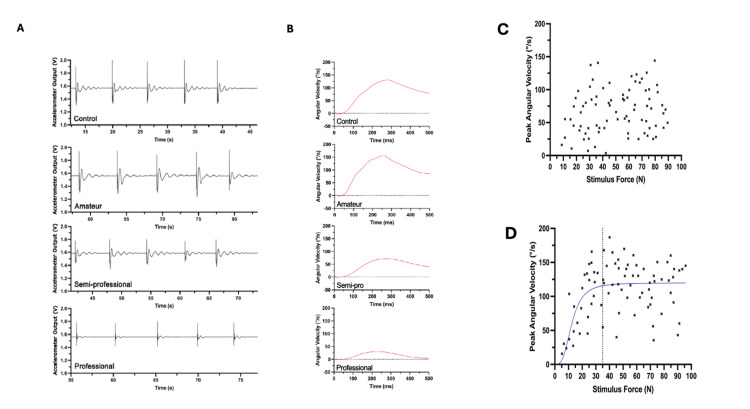
The raw acceleration traces, angular velocity, and response to stimuli graphs for each cohort of players Panel A: Example raw acceleration traces. One sample trace is shown for each of the control, amateur, semi-professional, and professional playing groups, with five reflex responses per group. Panel B: Angular velocity plots: A sample plot from each of the control, amateur, semi-professional, and professional groups. Angular velocity (ƈ/s) is plotted against time (ms) for a 500-ms period after peak stimulus force was detected. Saturation curve scatter plots: These graphs illustrate the relationship between stimulus force (in Newtons, N) and peak angular velocity (in degrees per second, °/s). Each data point represents an individual reflex response. Panel (C) shows the response to 80 stimuli, without consideration of reflex fatigue. Panel (D) displays 80 responses collected across eight recordings. The blue line is a sigmoidal function demonstrating reflex saturation, and the vertical dotted line indicates the threshold value used (35N).

The raw ankle acceleration traces were converted to angular velocity. A comparison between control, amateur, semi-professional, and professional subjects indicated a decrease in reflex magnitude with increased training exposure. The effects of rugby training level on reflex magnitude and asymmetry were analysed (Figure 3).

Reflex magnitude 

**Figure 4:**
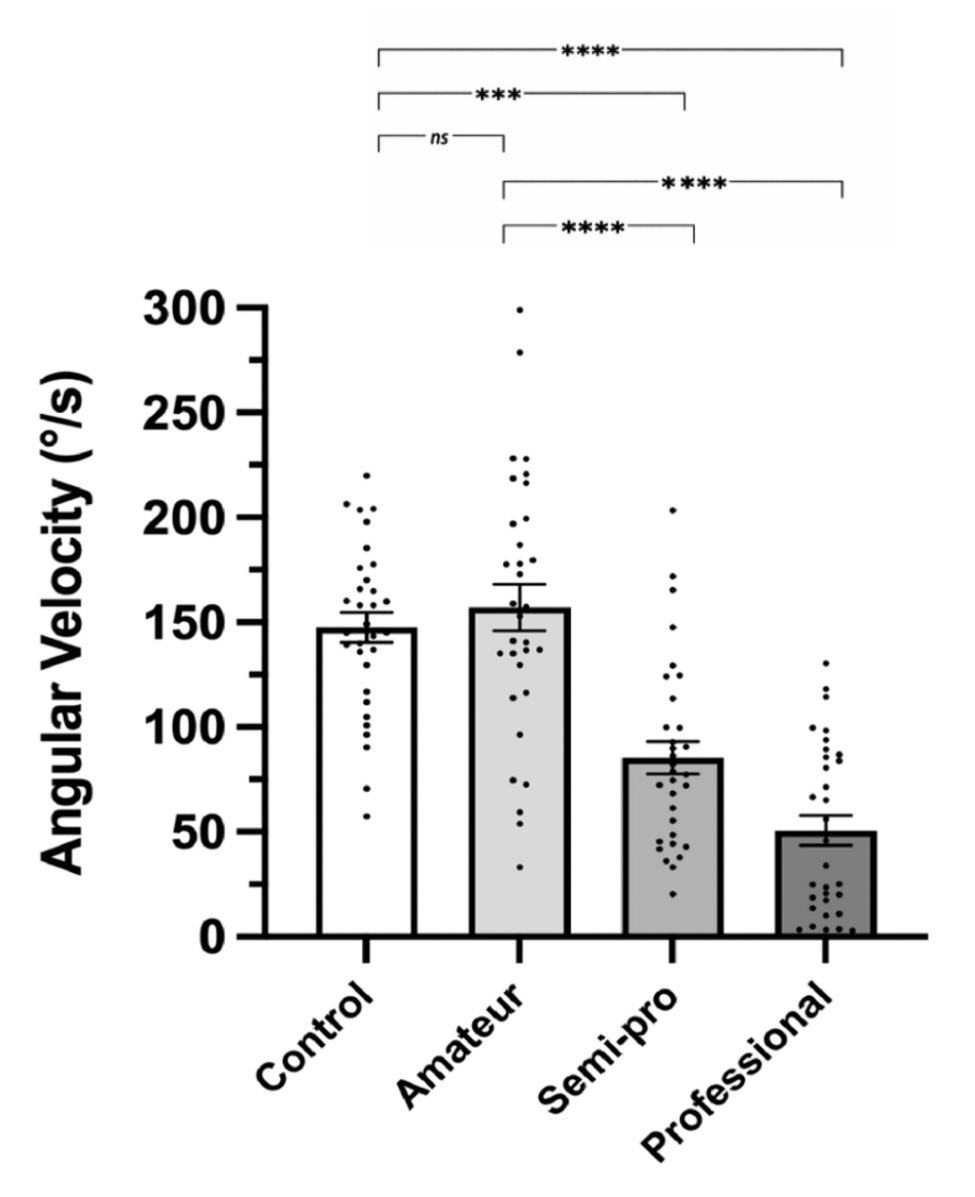
Mean angular velocity of each group Bar graph plotting the group mean angular velocity (°/s) of the top 10 reflexes from both legs of each subject within a group. Each data point represents an individual sample, and the error bars represent the SEM for each group. Significant results are denoted by ***  (p<0.001) and **** (p<0.0001). Non-significant results are denoted by ns. SEM: standard error of the mean

Our data were divided into groups: control, amateur, semi-professional, and professional (Table 2). The mean angular reflex velocity was plotted for all groups (Figure 4). We observed a progressive decrease as training increased. No significant difference was found between control and amateur (p=0.461). Significant differences were noted when comparing control and amateur with semi-professional and professional (p<0.0001). A significant difference was also observed between semi-professional and professional (p<0.001). 

**Table 2:** Summary statistics for each group The table displays the number of values, the mean, the percentage difference in the mean compared to the control, the minimum and maximum angular velocity values, and the SEM for each group: control, amateur, semi-professional, and professional. SEM: standard error of the mean

	Control	Amateur	Semi-professional	Professional
Number of values	32	32	32	32
Mean	147.5	157	85.4	50.7
% difference compared to control	-	+6.4%	-42.1%	-65.6%
Minimum	57.3	33.1	20.4	2.28
Maximum	219.8	298.8	203.4	130.4
SEM	7.046	11.09	7.797	7.133

Reflex asymmetry 

**Figure 5:**
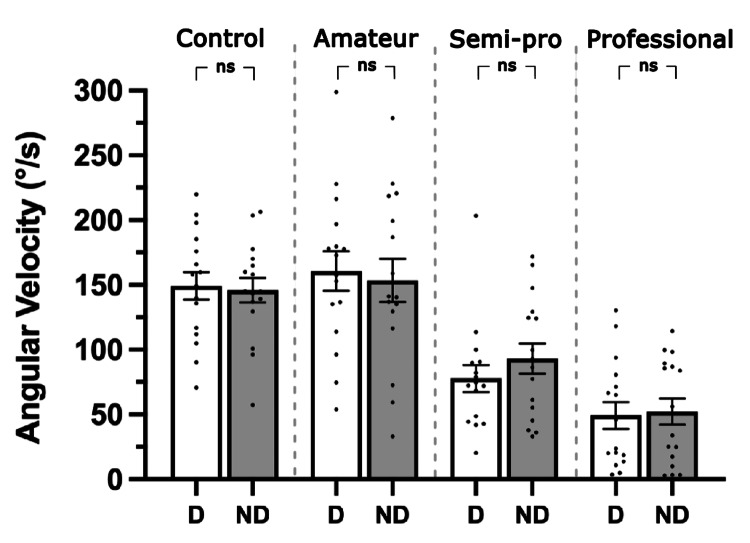
Bar graph of responses from dominant and non-dominant legs Mean angular velocity (°/s) of the top 10 reflexes in the dominant (D, white bars) and non-dominant (ND, grey bars) legs of subjects in each group. Each data point represents an individual sample. Error bars indicate SEM for each group. SEM: standard error of the mean

The data were divided by group and leg dominance to detect asymmetry between the dominant and non-dominant legs. Recordings from both legs were used to assess asymmetry (Figure 5). There was no evidence of asymmetrical responses (p=0.411). As data were taken from both legs of each subject, and thus all data were not independent, the effects of rugby playing level on reflex magnitude and asymmetry were investigated simultaneously using a two-way repeated-measures ANOVA. The dominant and non-dominant data were compiled, and the ANOVA model was used to test the effects of group, asymmetry, and their interaction (Table 3). Normal distribution and the absence of significant outliers were confirmed. The ANOVA indicated a significant group effect (p=3.18×10^-9^) but revealed no significant interaction (p=0.358) or difference between dominant and non-dominant legs (p=0.411). 

**Table 3:** Results of the two-way repeated-measures ANOVA The table shows the DFn and DFd, the F-value, the P-value, and the GES. The group effect (*) appears to be highly significant (p<0.0001). DFn: degrees of freedom for the numerator; DFd: denominator; GES: overall effect size

Factor	DFn	DFd	F	P	GES
Group	3	60	20.373	3.18×10^-9^*	0.470
Asymmetry	1	60	0.686	0.411	0.001
Group×asymmetry	3	60	1.095	0.358	0.007

Patellar reflex sensitivity 

**Figure 6:**
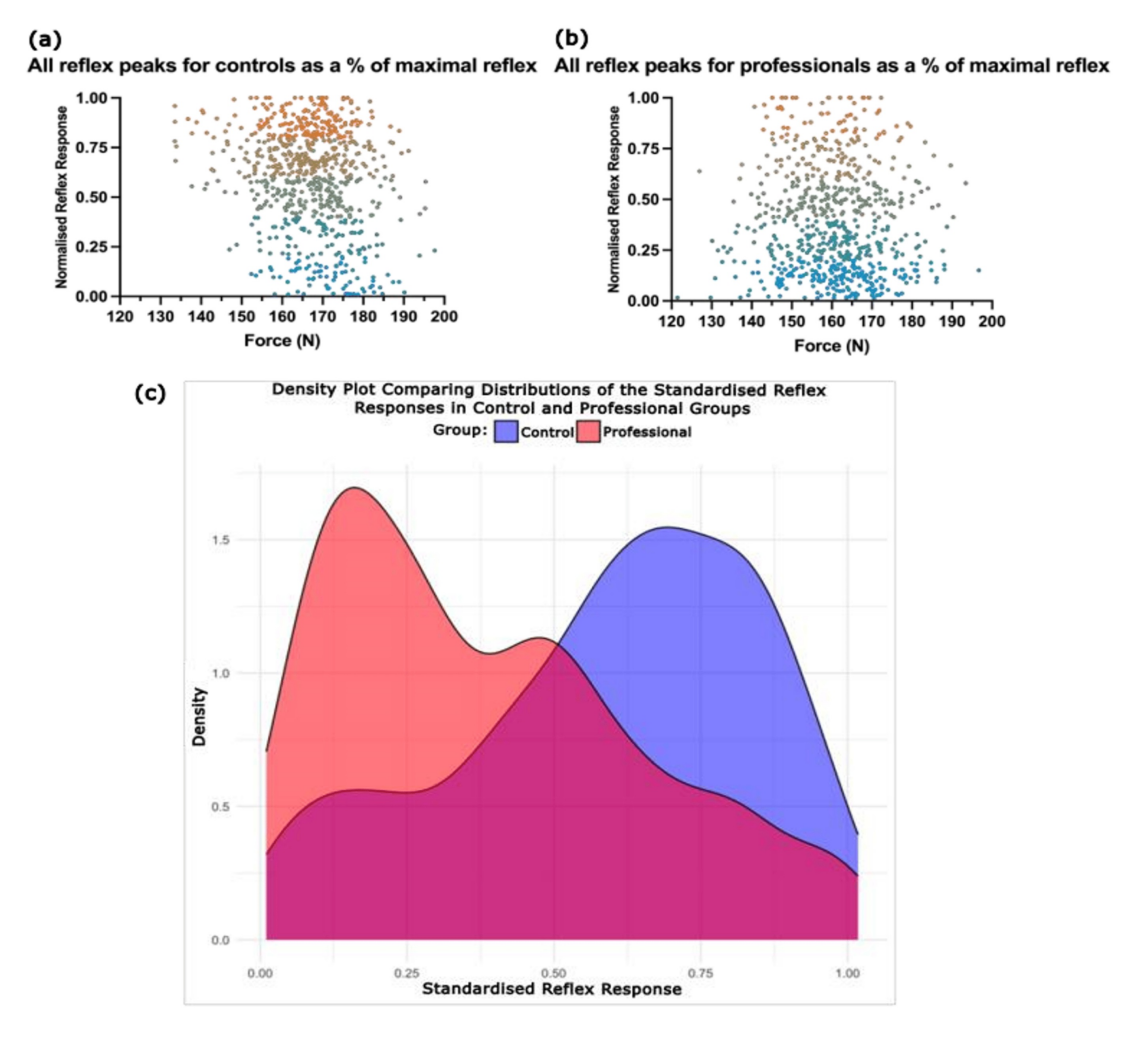
Standardised reflexes in the control group (a) and professional group (b) All the standardised reflex responses are plotted against stimulus force (N). Points are colour-coded to facilitate visual inspection with values 0-0.2 coloured blue, values 0.8-1 coloured orange, and intermediate values falling on the spectrum between the two. (c) Standardised reflex distributions. Density plot showing the distributions of the standardised reflex response between 0 and 1 of the control (blue) and professional (orange) groups.

To compare sensitivity to the stimulus between the control and professional groups, reflex angular velocity was standardised against the maximum reflex, producing a value from 0 (no reflex) to 1 (an individual's maximum reflex) (Table 4). Figure 6 displays scatter and density plots of these responses. A two-sample Kolmogorov-Smirnov test revealed a significant distributional difference (D=0.364; p=2.20×10¹⁶), indicating that the control group had more responses near the maximum, while the professional group clustered at lower responses. This demonstrates that eliciting maximum reflexes was less common in the professional group. 

**Table 4:** Backwards stepwise elimination by AIC output The minimal model is shown in the header of the table. <none> indicates the model without removing any factors. Each subsequent row displays the sum of squares, RSS, and AIC value when the relevant factor is eliminated from the model. Df: degrees of freedom; RSS: residual sum of squares; AIC: Akaike Information Criterion

	Angular velocity ~ playing level + calf circumference + age + concussion + playing level: concussion			
	Df	Sum of squares	RSS	AIC
	-	-	231943	980.28
Calf circumference	1	13885	245829	985.73
Age	1	14099	246042	985.84
Playing level: concussion	3	33270	265213	991.44

## Discussion

This study evaluates the impact of rugby training on the knee-jerk reflex using a novel, modified force transducer and accelerometer. The primary outcome was to examine whether playing level affected the magnitude of reflexes.

We observed a significant, progressive decrease in the magnitude of the patellar reflex with increased frequency of rugby training. Previous studies indicate that endurance sports enhance reflexes, whereas power sports tend to suppress them. This binary division needs to be reviewed, considering rugby's combined power and endurance demands. Elite rugby players cover long distances in matches, with short recovery periods between high-intensity efforts [24,25].

Thus, rugby straddles this binary division, requiring both power and endurance components. Considerations of metabolic demands and movement types between sports may provide greater insight into the basis of this reflex modulation. We identify three potential factors underlying the observed changes in reflex modulation: alterations in muscle composition, changes in synaptic transmission within the spinal cord, and variations in muscle spindle sensitivity. 

It is well-documented that exercise can alter muscle fibre composition [26]. In addition, slow-twitch (ST) fibres are known to contribute to reflex contraction [27]. Thus, a progressive shift from ST to fast-twitch (FT) fibres with increased rugby training, if present, could explain the observed reflex suppression [28-30]. Various training methods used in rugby, including sprint and resistance training, enhance FT and reduce ST fibres, respectively. This may lead to a decrease in the patellar reflex [31]. Noninvasive evaluations of fibre composition could support this possibility in future studies [32,33].

Reflex modulation can also be caused by neuromodulation of the reflex arc in the spinal cord. At the Ia-motoneuron synapse, presynaptic inhibition by GABAergic interneurons regulates sensory-motor drive, and local and descending inputs control the activity of these neurons [34]. These neurons modulate proprioceptive feedback, a critical determinant of motor stability [35]. The spinal cord constantly receives sensory and proprioceptive information from Ia afferents; however, to execute complex motor outputs, such as during rugby training, this input needs to be appropriately tempered to prevent unwanted oscillatory activity. At higher levels of rugby, the intensity of training and force of collisions increase. Therefore, augmented descending or local inhibition may contribute to the progressive suppression of the reflex observed in the present study. This may be a physiological adaptation that facilitates smooth motor output and reduces the risk of injury. 

Before a change in direction, athletes (such as rugby players) tense certain muscle groups, which serve a protective function by stabilising the joint, absorbing force, and reducing the risk of injury. This phenomenon is known as feed-forward co-contraction of heteronymous muscles [36-38]. Rugby players perform these co-contractions particularly during multidirectional movements, and these protective functions are relevant, for example, when changing direction at speed or bracing for collision [39]. Recurrent co-contractions of heteronymous muscles may contribute to reflex suppression through augmented presynaptic inhibition [40].

Another potential mechanism is a training-induced reduction in the muscle spindle gain. With decreased gain, spindles may be less responsive to a given change in muscle length, thereby reducing Ia excitation and suppressing reflex responses. In this study, an analysis of reflex sensitivity between control and professional subjects (Figure 6) supports the suggestion that reflex suppression is mediated by alterations in spinal cord spindle excitability and/or increased spinal cord inhibition. Given that the muscle fibre composition and motoneuron excitability remain unchanged between trials and that all stimuli were significantly above the threshold, the marked reduction in the proportion of maximal reflexes in the professional group compared to controls suggests the reflex was 'harder to evoke' (reduced spindle excitability) or that the Ia-⍺-motoneuron synaptic gain was reduced. Muscle stretching also decreases tendon reflex magnitude by reducing spindle sensitivity [41]. It is plausible that muscular compliance and reduced spindle gain may be adaptive, enabling fluent movements on a rugby pitch without disruptive reflexive contractions.

Limitations

This research introduced a new technique for measuring force and new equipment for acceleration quantification; however, it was not without limitations. Our transducer and accelerometer design enabled accurate reflex measurement. Although data processing is labour-intensive, processing the transducer signal with an embedded microcontroller could facilitate automated, real-time reflex classification, thereby enhancing device portability and its clinical or sports medicine applications [6]. The inter-stimulus interval was uncontrolled, and reflex fatigability was not accounted for. Better control would improve repeatability [42]. Moreover, there was no control over the other sports subjects played, and this study is insufficient to assess innate reflex differences that may have contributed to the results. While the progressive reduction of reflexes across groups is indicative of a training-induced effect, a longitudinal study tracking reflex changes in a single subject over time would help further dissect these factors. 

Future work

Incorporating training frequency and duration data into future analysis may enable the estimation of 'training age'. This may offer further insight into changes in reflex magnitude. Testing at different times of day and a lack of control over muscle stretching may have also contributed to variability [41,43]. Moreover, this study was confined to a single sport. Comparative studies of similar sports could uncover which training methods and movements contribute to this suppression. For example, comparing elite football and rugby players might clarify whether reflex reduction results from the high-speed, explosive movements common to both sports or from the requirement for stability against externally applied forces, which is more specific to rugby. The incidence of unexplained anterior cruciate ligament ruptures in elite female athletes is three to five times higher than in male athletes. While likely multifactorial, including valgus knee alignment, lower knee stiffness, and greater anterior tibial laxity, extending the current study to include female rugby players could provide valuable insights into their increased injury risk [44,45]. In addition, the relationship between reflex modulation and anterior cruciate ligament injury has been previously examined in the context of anthropogenic muscle inhibition, an area that warrants further investigation [46].

## Conclusions

By developing a novel force transducer and accelerometer, this study presents an accessible and accurate method for quantifying reflexes, which, with further development, may become a clinical avenue of interest. This study demonstrated a gradual diminution in patellar reflex responses with increased exposure to rugby training. This indicates training-dependent reflex adaptation, which may result from central and/or peripheral plasticity. Overall, a combination of factors likely causes the reflex changes observed with rugby training. However, a better understanding of these effects could help clinicians better understand injuries among athletes of both sexes across various sports and provide additional detail during clinical examinations. 

## Figures and Tables

**Figure 7:**
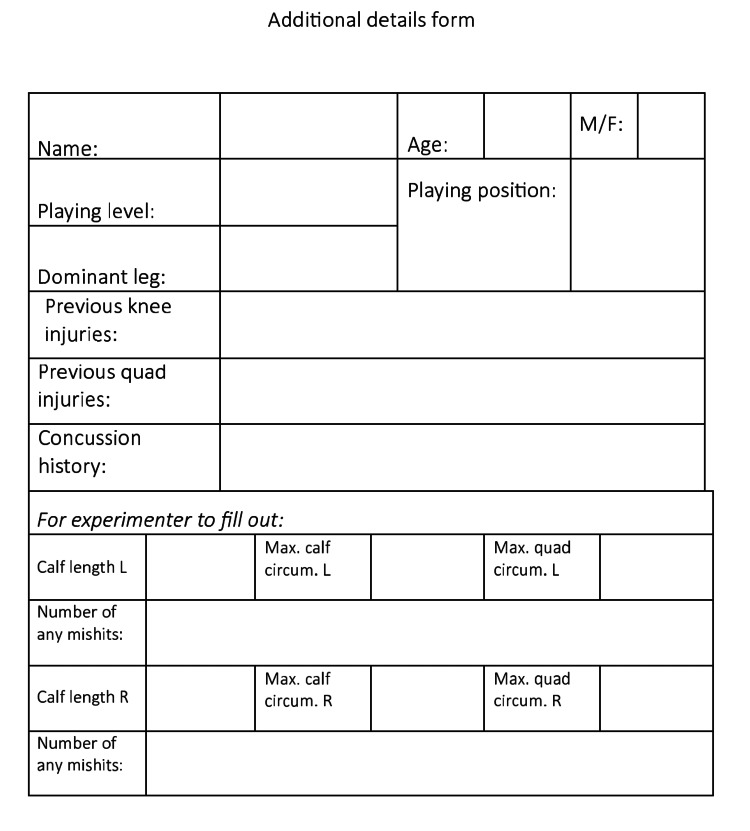
Participant questionnaire
